# Predicting poverty. Data mining approaches to the health and demographic surveillance system in Cuatro Santos, Nicaragua

**DOI:** 10.1186/s12939-019-1054-7

**Published:** 2019-10-29

**Authors:** C. Källestål, E. Blandón Zelaya, R. Peña, W. Peréz, M. Contreras, L. Å. Persson, O. Sysoev, K. Ekholm Selling

**Affiliations:** 1 Department of Women’s and Children’s Health, Uppsala University, Uppsala, Sweden; 2 Asociación para el Desarrollo Económico y Sostenible de El Espino (APRODESE), Chinandega, Nicaragua; 3 UNAN-León, León, Nicaragua; 4 Department of Computer and Information Science, Linköping University, Linköping, Sweden; 5 Department of Disease Control, London School of Hygiene & Tropical Medicine, London, UK; 6 Pan American Health Organization, Tegucigalpa, Honduras

**Keywords:** Poverty, Prediction, Conditional inference trees, Conditional random forest analyses, Datamining, Education

## Abstract

**Background:**

In order to further identify the needed interventions for continued poverty reduction in our study area Cuatro Santos, northern Nicaragua, we aimed to elucidate what predicts poverty, measured by the Unsatisfied Basic Need index. This analysis was done by using decision tree methodology applied to the Cuatro Santos health and demographic surveillance databases.

**Methods:**

Using variables derived from the health and demographic surveillance update 2014, transferring individual data to the household level we used the decision tree framework Conditional Inference trees to predict the outcome “poverty” defined as two to four unsatisfied basic needs using the Unsatisfied Basic Need Index. We further validated the trees by applying Conditional random forest analyses in order to assess and rank the importance of predictors about their ability to explain the variation of the outcome “poverty.” The majority of the Cuatro Santos households provided information and the included variables measured housing conditions, assets, and demographic experiences since the last update (5 yrs), earlier participation in interventions and food security during the last 4 weeks.

**Results:**

Poverty was rare in households that have some assets and someone in the household that has a higher education than primary school. For these households participating in the intervention that installed piped water with water meter was most important, but also when excluding this variable, the resulting tree showed the same results. When assets were not taken into consideration, the importance of education was pronounced as a predictor for welfare. The results were further strengthened by the validation using Conditional random forest modeling showing the same variables being important as predicting the outcome in the CI tree analysis. As assets can be a result, rather than a predictor of more affluence our results in summary point specifically to the importance of education and participation in the water installation intervention as predictors for more affluence.

**Conclusion:**

Predictors of poverty are useful for directing interventions and in the Cuatro Santos area education seems most important to prioritize. Hopefully, the lessons learned can continue to develop the Cuatro Santos communities as well as development in similar poor rural settings around the world.

## Background

The first of the Sustainable Development Goals aims at ending poverty in all its forms, everywhere [1]. Poverty is measured by the World Bank and many international agencies as monetary measures on the national level, such as the poverty line at 1.90 purchasing power parity dollar and the Gross Domestic Product per capita measures. These monetary measures of poverty are possible to compare over time and across nations. In Latin America the Unsatisfied Basic Need (UBN) index has been widely used to compare poverty at the household level in different geographical areas [2, 3]. UBN is a composite index that includes housing conditions, access to water and sanitation, school enrolment, education of the head of household, and the ratio of dependent household members to working age members. In the Demographic Health Surveys (https://www.dhsprogram.com) asset scores have been widely used as measurement of household socioeconomic status and poverty [4]. Asset scores have been used to stratify other outcomes along a wealth axis, such as the identification and explanation of social inequalities in health [5]. These scores cannot be used to follow or compare development over time since each index is only valid for the survey for which it was created.

Governments have the responsibility to implement policies for poverty reduction to reach the first Sustainable Development Goal [6]. Local-level bottom-up interventions might, however, result in sustainable poverty reduction that can inspire decision makers at the national level. We have documented such a case from northern Nicaragua; the Cuatro Santos experiences of local poverty reduction [7]. That case study showed that in addition to a bottom-up approach, factors such as local ownership, locally guided multidimensional interventions, and close monitoring and evaluation of the development efforts yielded a substantial poverty reduction of household poverty from 79 to 47% over 10 year (2004–14) [7].

In the Cuatro Santos area, a Health and Demographic Surveillance System (HDSS) was established in 2004 with the latest update in 2014. Participation in microcredit programs, the involvement of young individuals in technical training, and home gardening were all associated with the transition of households out of poverty [8]. The Unsatisfied Basic Need scoring of households was used to identify geographic areas with higher levels of poverty to target interventions [7].

In order to further identify the needed interventions for continued poverty reduction, we wanted to elucidate what predicts poverty, measured by the Unsatisfied Basic Need index, in this setting. The common epidemiological and statistical methods have limitations in approaching these kinds of wide research questions, i.e. research questions investigating a large number of potentially important variables in relation to one outcome, with potentially complex and multiple interactions between the predictors. Thus, a decision-tree methodology framework called conditional inference (CI) trees was used. CI trees is a modern type of decision-trees, which allows for specifying an arbitrarily high number of predictor variables, handling variables of different types, automatically discovering complex interactions between predictor variables, and including them into the model [9].

## Methods

### Aim

The aim of this paper was to identify predictors of poverty measured as unsatisfied basic need by using decision tree methodology to the Cuatro Santos health and demographic surveillance databases, Nicaragua.

### Study setting, population, and design

**Fig. 1:**
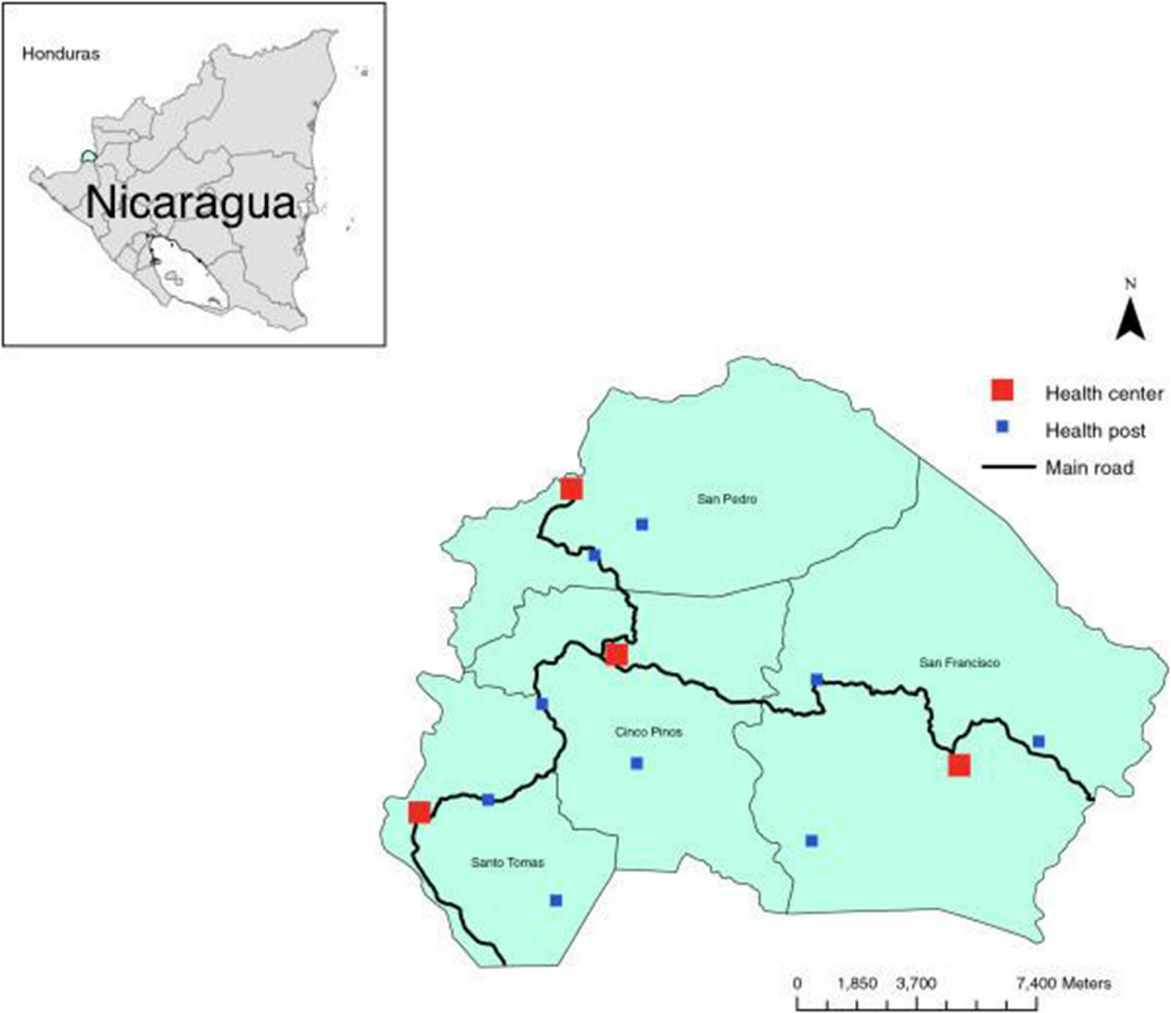
The Cuatro Santos area showing the four municipalities and health facilities. The area is marked in the inserted Nicaragua map

The Cuatro Santos area, situated in the northern part of Chinandega, Nicaragua, consists of four municipalities of similar population size. In 2014 totally 25,893 inhabitants lived in 5966 households (Fig. 1). The area is located 250 km northwest of the capital of Nicaragua, Managua, in a mountainous terrain bordering Honduras. The climate is predominantly dry and the traditional source of income has been the cultivation of grains and raising livestock, now with an increasing number of small-scale enterprises. This area was strongly affected by the Contras war in the 1980s and the hurricane Mitch in October 1998. Since that time, a significant proportion of the population has out-migrated due to economic reasons (including fixed or seasonal work or search for employment) [10].

### Community interventions in Cuatro Santos

A process labeled “decoding reality”, inspired by Paulo Freire [11] were initiated 1997 by representatives of the four municipalities, the local non-governmental organizations, local government leaders, and representatives of national institutions. This process included an analysis of the local poverty-related problems, prioritization among suggested actions, and an action plan that was approved as the Cuatro Santos Area Development Strategy for the period 2002 to 2015. This strategy aimed at efforts to develop the area by use of local resources, informed by data from the surveillance system, and actions to attract international cooperation. The concepts of local ownership and participation were central, and the efforts included consensus decision-making and reconciliation in case of conflicts. Priority interventions were school breakfasts, environmental protection, water and sanitation, preventive healthcare, home gardening, microcredits, technical training, stipends for university education, and telecommunications including access to and training to use the Internet. Data collection through a Health and Demographic Surveillance System (HDSS) was central for monitoring of trends over time, and research evaluation of various aspects [7, 8].

### Cuatro Santos health and demographic surveillance system

In 2004, a census and cross-sectional data collection of basic health and demographic information was performed in the whole population. Follow-up surveys were performed in 2007, 2009, and 2014 and unique identifiers of households and individuals linked the data. Demographic changes in households, such as birth, death, and migration, were registered. Household data included information on the house (floor, walls) and services (water, sanitation, electricity). All women aged 15–49 years living in households provided retrospective reproductive histories [8]. In the 2009 and 2014 updates, questions were included on participation in the following interventions: access to water and latrines, microcredit, home gardening, technical education, school breakfast programs, and telecommunications. In the 2014 update data on food security, household assets, and women’s self-rated health were also collected. For the present study, data from the 2014 update including data on earlier experienced events e.g., participation in interventions were used.

Local women with at least high school education were trained by the researchers and the field supervisor to form the group of fieldworkers engaged in all updates of the HDSS. Refreshment training has been conducted at the start of every update. Fieldwork was carefully supervised, forms were checked before computerization, and the forms were returned to the field if the information was missing or suspected to be incorrect. Further quality controls were completed after computerization including logical controls. Data were carefully cleaned and stored in a relational database.

### Variables (Table 1)

Persons residing in a household at the time of the field survey defined the household. Migration was defined as a household member aged 18–65 who migrated in or out of the household since the latest update (5 yrs.). The Unsatisfied Basic Need index [2] was composed by four components: (1) housing conditions (unsatisfied: walls of wood, cardboard, plastic or earthen floor); (2) access to water and latrine (unsatisfied: water from river, well, or bought in barrels and no latrine or toilet); (3) school enrolment of children (unsatisfied: any children 7–14 years of age not attending school); and (4) education of head of the family and ratio of dependent (< 15 yrs. and > 65 yrs.) household members to working age members (15–65 yrs.) (unsatisfied: head of the family illiterate or dropped out of primary school and ratio of dependent household members to working age members. > 2.0). Each component rendered a score of zero, if satisfied, and one, if unsatisfied. Thus, the total sum varied from zero to four. Households with zero or one unsatisfied basic need were considered non-poor, while poor households had two to four unsatisfied basic needs. Characteristics of houses and households were also included in the analyses, such as access to electricity, and type of stove. The interventions implemented in the area were represented by household-related information on such participation. The presence of a water meter indicated that the household had got water installed as part of the last decade’s interventions. Also, information was included on previous and current participation in home gardening, if anyone in the household had received microcredit or had participated in technical training.

**Table 1:** Variables list for the analyses of Cuatro Santos database, Nicaragua 2014, including descriptive statistics

Categorical variables	In analysis^b^	Labels	Number	Percent
Poverty	Outcome	0 Not poor = UBN 0–1	2828	53.8
	Outcome	1 Poor = UBN 2–4	2425	46.2
Unsatisfied Basic Need (UBN)	Base for outcome	0 No basic need unsatisfied	1161	22.1
	Base for outcome	1 Wall is made of wood, cartons, plastic AND mud floor	1667	31.7
	Base for outcome	2 Access to water is through rivers, wells, or bought in barrels AND no latrine	2167	41.3
	Base for outcome	3 Children ages 7 to 14 years are not attending school	251	4.8
	Base for outcome	4 The head is illiterate or not completed primary school AND dependency ratio > 2	7	0.1
House wall type	Excluded	1 Ceramic brick	1465	27.9
		2 Adobe/wattle wall	3707	70.6
		3 Wood	31	0.6
		4 Palm	3	< 0.1
		5 Cardboard, Plastic, Metal	42	0.8
		6 Without walls	5	< 0.1
Water availability	Excluded	1 Inside pipe	1807	34.4
		2 Commune post	117	2.2
		3 Own well	1117	21.3
		4 Communal well	1538	29.3
		5 River/Creek	410	7.8
		6 Purchased water	6	0.1
		7 Other sources	258	4.9
Toilet type	Excluded	1 Toilet	133	2.5
		2 Latrine	4123	78.5
		3 No toilet/latrine	997	19.0
Floor in house	Excluded	1 Ceramic brick	418	8.0
		2 Brick/cement	272	5.2
		3 Mud brick	42	0.8
		4 Tiling	1567	29.8
		5 Mud floor	2954	56.2
Electricity in house	Excluded in 2nd	1 Yes	4683	89.1
		2 No	570	10.8
Stove in house	Excluded in 2nd	1 Gas	469	8.9
		2 Wood/improved	75	1.4
		3 Wood/normal	4664	88.8
		4 Does not have	45	0.9
Water meter in use	Excluded in 2nd	1 Yes	1130	21.5
		2 No	4123	78.5
Microcredit in HH^a^	Used	1 Yes	671	12.8
		2 No	4582	87.2
Technical training in HH^a^	Used	1 Yes	514	9.8
		2 No	4739	90.2
Home garden in HH^a^	Used	1 Yes	321	6.1
		2 No	4932	93.9
Home garden in use	Used	1 Yes	197	3.8
		2 No	5056	96.2
Anxiety in HH^a^ for lack of food	Used	0 Never	705	13.4
		1 Rarely (1–2 times)	2106	40.1
		2 Sometimes (3–10 times)	1303	24.8
		3 Often (> 10 times)	1139	21.7
Inability in HH^a^ to eat preferred food	Used	0 Never	692	13.2
		1 Rarely (1–2 times)	2216	42.2
		2 Sometimes (3–10 times)	1803	34.3
		3 Often (> 10 times)	542	10.3
Limited variation of food in HH^a^ due to lack of food	Used	0 Never	989	18.8
		1 Rarely (1–2 times)	2421	46.1
		2 Sometimes (3–10 times)	1440	27.4
		3 Often (> 10 times)	403	7.7
Few kinds of food consumed in HH^a^ due to lack of food	Used	0 Never	896	17.1
		1 Rarely (1–2 times)	2584	49.2
		2 Sometimes (3–10 times)	1427	27.2
		3 Often (> 10 times)	346	6.6
Reduction of portion sizes of meals in HH^a^ due to lack of food	Used	0 Never	1307	24.9
		1 Rarely (1–2 times)	2524	48.0
		2 Sometimes (3–10 times)	1166	22.2
		3 Often (> 10 times)	256	4.9
Fewer meals consumed in HH^a^ due to lack of food	Used	0 Never	2016	38.4
		1 Rarely (1–2 times)	2167	41.3
		2 Sometimes (3–10 times)	892	17.0
		3 Often (> 10 times)	178	3.4
No food to eat in HH^a^ due to lack of resources	Used	0 Never	3734	71.1
		1 Rarely (1–2 times)	1132	21.5
		2 Sometimes (3–10 times)	335	6.4
		3 Often (> 10 times)	52	1.0
HH^a^ going to sleep hungry due to lack of food	Used	0 Never	4478	85.2
		1 Rarely (1–2 times)	564	10.7
		2 Sometimes (3–10 times)	189	3.6
		3 Often (> 10 times)	22	0.4
HH^a^ having days of hunger due to insufficient amount of food	Used	0 Never	4744	90.3
		1 Rarely (1–2 times)	367	7.0
		2 Sometimes (3–10 times)	124	2.4
		3 Often (> 10 times)	18	0.3
TV antenna in HH^a^	Excluded in 2nd	1 Parabolic antenna	604	11.5
		2 Normal antenna	2069	39.4
		3 Handmade antenna	429	8.2
		4 No antenna	2151	40.9
Car in HH^a^	Excluded in 2nd	1Yes	137	2.6
		2 No	5116	97.4
Motorbike in HH^a^	Excluded in 2nd	1Yes	443	8.4
		2 No	4810	91.6
Bike in HH^a^	Excluded in 2nd	1Yes	872	16.6
		2 No	4381	83.4
Horse in HH^a^	Excluded in 2nd	1Yes	1347	25.6
		2 No	3906	74.4
Refrigerator in HH^a^	Excluded in 2nd	1Yes	1567	29.8
		2 No	3686	70.2
Sewing machine in HH^a^	Excluded in 2nd	1Yes	337	6.4
		2 No	4916	93.6
Computer in HH^a^	Excluded in 2nd	1Yes	183	3.5
		2 No	5070	96.5
Tortilla oven in HH^a^	Excluded in 2nd	1Yes	916	17.4
		2 No	4337	82.6
Stove with chimney in HH^a^	Excluded in 2nd	1Yes	103	2.0
		2 No	5150	98.0
Deaths in HH^a^	Used	0 No deaths in HH^a^	4934	93.9
		1 Deaths in HH^a^	319	6.1
Births in HH^a^	Used	0 No births in HH^a^	3907	74.4
		1 Births in HH^a^	1346	25.6
Immigration in HH^a^	Used	0 No immigration in HH^a^	3206	61.0
		1 Immigration in HH^a^	2047	39.0
Emigration in HH^a^	Used	0 No emigration in HH^a^	2289	43.6
		1 Emigration in HH^a^	2964	56.4
Sex of HH head	Used	1 Female head of HH^a^	1382	26.3
		2 Male head of HH^a^	3871	73.7
Illiterate living in HH^a^	Used	0 No illiterate in HH^a^	3812	72.6
		1 Illiterate in HH^a^	1441	27.4
Highest education in HH^a^	Used	0 No education	208	4.0
		2 Primary school	1679	32.0
		3 Secondary school	2312	44.0
		4 Technical education	379	7.2
		5 University education	675	12.8
HH^a^ member immigrated from foreign country	Used	0 No immigration from other country in household	4928	93.8
		1 Immigration from other country in HH^a^	325	6.2
HH^a^ member emigrated to foreign country	Used	0 No emigration to other country in HH^a^	4560	86.8
		1 Emigration to other country in HH^a^	693	13.2
Child/ren (< 15 yrs.) In HH^a^ working	Used	0 No	5172	98.4
		1 Yes	81	1.5
Home birth in HH^a^	Used	0 No home birth in HH^a^	5143	97.9
		1 Home birth in HH^a^	110	2.1
Hospital birth in HH^a^	Used	0 No hospital birth in HH^a^	4153	79.1
		1 Hospital birth in HH^a^	1100	20.9
Child health center birth in HH^a^	Used	0 No CHC birth in HH^a^	4892	93.1
		1 CHC birth in HH^a^	361	6.9
Women’s self-rated health in HH^a^	Used	0 No women with bad health in HH^a^	2963	56.4
		1 Women with bad health in HH^a^	2290	43.6
	Continuous variables			
		Mean (Median)	Min	Max
No of children in HH^a^	Used	1.7 (2.0)	0	12
No of adults in HH^a^	Used	4.7 (4.0)	0	19
No in HH^a^ not working	Used	2.6 (2.0)	0	13
No in HH^a^ working	Used	1.4 (1.0)	0	9
No of working adults (> = 15 yrs.) In HH^a^	Used	1.4 (1.0)	0	9
No of not working adults (> = 15 yrs.) In HH^a^	Used	1.7 (1.0)	0	8
No of individuals in HH^a^	Used	6.5 (6.0)	1	25
Ratio of adults working to not working in HH^a^	Used	1.6 (1.0)	0	9
Ratio of working adults (> = 15 yrs.) To no of individuals in HH^a^	Used	0.2 (0.2)	0	1

^a^*HH* household, ^b^Used means used in both CI tree analyses. Excluded due to variable included in Unsatisfied Basic Need index. Excluded in 2nd means included in first analysis and excluded in second analysis due to being an asset

A nine-item Household Food Insecurity Access Scale (HFIAS), version 3, was used [12]. The respondents were either the head of the household or the person responsible for the household expenditure and food preparation. This scale covers experiences regarding 1) anxiety in the household due to lack of food; 2) inability to eat preferred food because of lack of resources; 3) limited variety of food due to lack of resources; 4) consumption of few kinds of food because of lack of resources; 5) reduction of portion sizes of meals due to lack of food; 6) consumption of fewer meals per day because of lack of food; 7) no food to eat in the household because lack of resources; 8) going to sleep at night hungry due to lack of food, and 9) days of hunger because of insufficient amounts of food to eat. For each affirmative answer, the person provided additional information on the frequency in a four-point scale (never, rarely, sometimes, often).

Household assets were TV antenna, car, motorbike, bike, horse, refrigerator, sewing machine, computer, tortilla oven, and a chimney for the wood-burning stove.

The individual variables were derived and aggregated to the household level, and then merged with the variables originally at the household level. We constructed variables on births and deaths in the household during the recent update period, also including information number of adults and children living in the household, number of adults and children working, number of adults not working, and the ratio between adults working and not working, as well as the ratio between adults working and number of individuals in the household. Further, data were included on in- and out-migration, including from foreign countries, the gender of household head, any illiteracy, and the highest education level in the household (none, primary, secondary, technical, university education). Information was also included if a home-, health center-, or hospital birth had happened since the last update (5 yrs).

Women’s self-rated health was assessed for all resident women of reproductive age (15–49 years) at time of the interview by a five-point Likert scale based on the following question: “In general, how would you assess your health today?” The interviewer provided the following options: very good, good, medium, bad, or very bad. This information was classified as good (very good, good, medium) or bad (bad, very bad) health. No household had a mix of good and bad self-assessed health when aggregating this information to the household level.

### Analytical methods

**Fig. 2:**
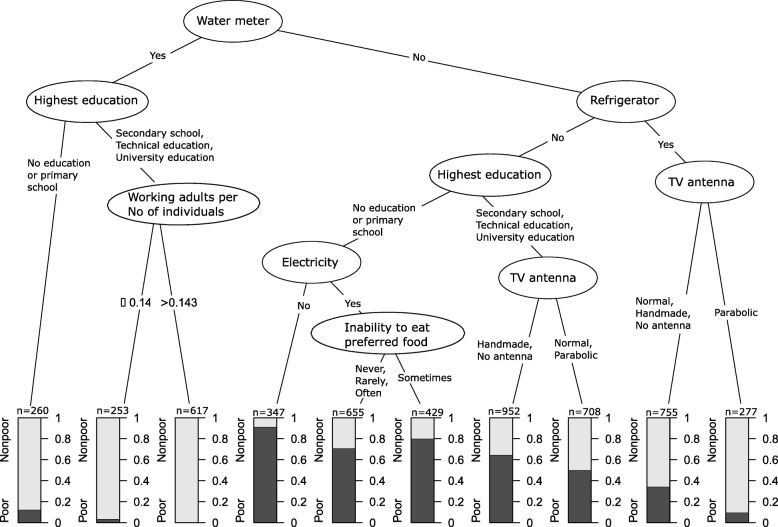
Cross-validated conditional inference tree with variables in the unsatisfied basic need index excluded. Each end node includes at least 250 individuals. Black areas in end nodes show proportions of poor (2–4 unsatisfied basic needs) and grey areas non-poor (0–1 unsatisfied basic needs). All variables measured at household level

**Fig. 3:**
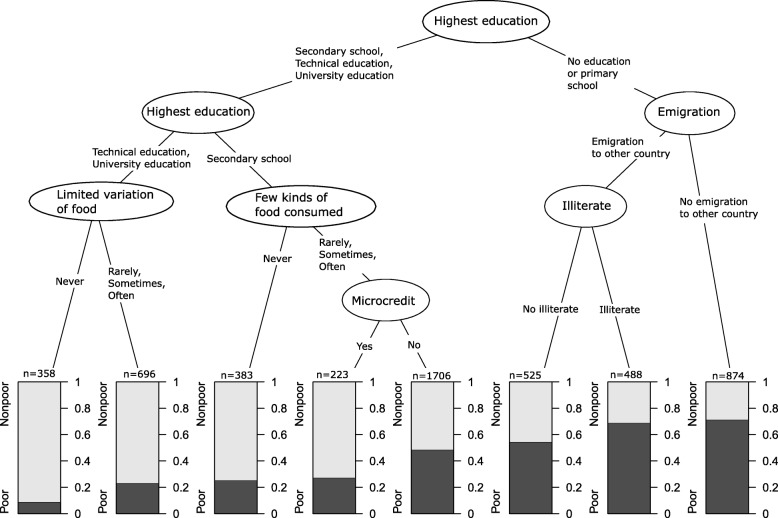
Cross-validated conditional inference tree with candidate predictor variables on assets and part of unsatisfied basic need index excluded. Each end node is at least 200 individuals. Black area in end nodes shows proportion of poor (2–4 unsatisfied basic needs) and grey non-poor (0–1 unsatisfied basic needs). All variables measured at household level

All analyses were performed on the household level. The variables included in the analyses are displayed in Table 1. Conditional Inference (CI*)* trees is one of the recent decision tree frameworks used in data mining that allows for specifying an arbitrarily high number of predictor variables, handling variables of different types, automatically discovering complex interactions between predictor variables, and including them into the model [9, 13]. The CI tree method embeds a statistical hypothesis-testing framework into a recursive partitioning algorithm for model building [13]. In this study, CI trees were used to identify subgroups characterized by combinations of levels of certain predictors with distinct values of the outcome” poverty” (defined as two to four unsatisfied basic needs). The number of candidate predictors evaluated for inclusion was 49 (Table 1) as variables included in UBN were excluded (Fig. 2). When all variables measuring assets were excluded the number of candidate predictors was 36 (Table 1 and Fig. 3). Cross-validation, a well-established model selection method was applied to select the tree of optimal size and the best predictive performance [14]. To ensure public health relevance, the minimum number of observations in each terminal node (subgroup) was set to 200 and 250. To further validate the obtained trees, we applied Conditional Random Forest (CRF) analyses in order to assess and rank the importance of predictors with regard to their ability to explain the variation of the outcome” poverty”. In conditional random forest analysis, an ensemble of conditional inference trees is created by through drawing subsamples from the original data and estimating a randomized conditional inference tree from each sample. Possible predictors at each split are selected randomly from the complete set of predictors, which leads to a better predictive performance of the tree ensemble [14]. The importance of a variable is computed by comparing the predictive mean squared error (MSE) from the original data and from a dataset where the corresponding variable values are specified incorrectly, If the variable is not important the difference between the original data MSE and the permuted data MSE should be relatively small. Therefore, an aggregated difference between the MSE values over the given ensemble of trees makes up the relative importance of a variable. The random forests analyses were created based on 1000 trees, and the ten variables with the highest importance measure are presented. Programming language R version 3.2.4 [15] and the “party” package [16] were used for all analyses.

## Results

Of the 5966 households included in the 2014-update of the HDSS, 5253 (88%) were included in the following analyses after eliminating households with missing values on any variable, the major part being houses not used as living quarters such as schools, health centers, abandoned houses, etc., that had been incorrectly included in the database as households. Included variables measured women’s self-rated health at the time of the interview, food security during the last 4 weeks, housing conditions, assets and demographic experiences since the last update (5 yrs) and earlier participation in interventions. Overall, 54% of households were classified as poor defined as two to four unsatisfied basic needs according to the Unsatisfied Basic Need index. Characteristics of the households are shown in Table 1.

### CI tree analysis

In the CI tree analysis, using 49 predictor variables (Table 1) the presence of piped water with water meter was the most important splitting variable in the tree shown in Fig. 2, followed by the highest education level in household and ownership of a refrigerator. The subgroup of households with the least likelihood of poverty (*n* = 617) were those who had piped water with a water meter in use, with at least someone in the household who had secondary education or higher and had a ratio of working adults to individuals in household higher than 0.143. In contrast, the subgroup of households in which poverty was most pronounced (*n* = 347) consisted of those with no water meter, no refrigerator, with the highest level of education among household members being primary school, and without electricity.

In households that had not participated in interventions (water meter) or had modern assets (refrigerator) the proportions of poor were lower in subgroups with higher educational level (nodes 7 & 8 from left).

The Conditional random forest analysis (Additional file 1: Figure S1) made in order to further validate the predictors present in the tree and with the same variables included showed that the three most important predictors coincided with the findings in the CI tree analysis with participation in the piped water with water meter intervention, having a refrigerator and the household’s highest education level being most important. These variables were followed by other assets not being predictors in the CI tree analysis (TV antenna, motorbike and horse in household) while also having electricity in the house, stove with a chimney and having participated in the microcredit intervention came among the most important ten predictors and coincided with the predicting variables in the CI tree analysis. Finally, having an illiterate member in the household was the tenth variable in importance (Additional file 1: Figure S1). As water meter is a subset to one of the variables in the Unsatisfied Basic Need index (access to water) we performed a sensitivity analysis excluding the variable water meter, and the resulting tree showed the same remaining variables and splits as before, even if water meter was excluded.

When assets were removed as candidate predictors, and 36 predictors (Table 1) remained in the analysis, the importance of education was even more evident (Fig. 3). The highest household education level differentiated between those nodes with higher and lower proportions in poverty, further strengthened by predicting higher proportion of poverty when someone in the household is illiterate. Also, in the validation using Conditional random forest analysis (Additional file 2: Figure S2) the variable highest household education level was the far most important with the other variables found in the CI tree predicting poverty appearing with less importance (microcredit, illiterate in household and several indicators of food insecurity). Only the last two variables in importance order did not coincide with variables found in the CI-tree analysis (number of adults not working in the household and household member that emigrated).

## Discussion

Poverty was rare in households that participated in the intervention that installed piped water with a water meter, have some assets and someone in the household that has a higher education than primary school. When assets were not taken into consideration, the importance of education was pronounced as a predictor for welfare.

As we cannot know the temporal relation between poverty and assets, assets can be a result, rather than a predictor of more affluence. The results of the CI tree analysis were further strengthened by the validation using Conditional random forest modeling showing the same variables being important as predicting the outcome in the CI tree analysis. Thus, in summary, our results point specifically to the importance of education and participation in the water installation intervention as predictors for more affluence.

In order to reduce the possible collinearity between variables constituting the Unsatisfied Basic Need index that was used to construct our outcome variable poverty, and our predictor variables, we eliminated coinciding variables (house wall type, water availability, toilet type and floor in the house) as candidate predictors. The two candidate predictor variables included that measure household education (illiterate living in the household and highest education in the household) should not cause collinearity as the educational variables included in the Unsatisfied Basic Need index showed to be present in less that 5% of the households (Table 1). The sensitivity analysis excluding the variable water meter that is a subset of the Unsatisfied Basic Need index’s part measuring access to water made no difference to the resulting tree. Still, the role of the intervention ‘water meter’ in relation to the outcome should be interpreted with some caution.

The Health and Demographic Surveillance data have shown to be of high quality [7, 8] and covers the whole population in the Cuatro Santos area with very few non-participants, thus providing a reliable source for analyzes. The temporality of poverty predictors (predictor happen before poverty) is not fully captured with our design, however by using dates for when interventions were commenced, stored in our database, we can state that most interventions happened before the 2014 update. When in time assets were acquired in relation to the time for the field survey we do not know, nor do we know when the head of the household was established but we have run analyses (not shown here) showing stability over time of head of household. For food insecurity the answers covered experiences during the last 4 weeks. Food insecurity is still at a high level in the area, although reduced over the last 5 years. The proportion of severely food insecure households dropped from 36% in 2009 to 29% in 2014 [17].

Using decision-tree-based methods such as CI tree enabled us to include simultaneously, and assess the importance of, a relatively large set of predictor variables on the outcome poverty, but also to include and evaluate interactions between the predictors automatically. The output from a CI tree analysis further displays precise information about the direction, size, and priority of the found associations. The purpose of the CI tree analyses was to find important predictors and their interactions rather than predicting the poverty status of an individual. Accordingly, traditional classification prediction metrics like misclassification rate have no relevance in our context and are not reported. However, since we have selected the depth of the decision trees by the cross-validation, the selected CI trees have the optimal predictive performance among feasible CI trees and the selected trees are not under- or overfitted to the training data. The actual quality of the models can thus be investigated by observing the impurity (probabilities) of the tree nodes. For example, Fig. 3 suggests that the tree has high quality because the probabilities in the tree leave that in most cases are far from an uninformative probability 0.5.

If we had used classical regression models, it would be impossible to estimate the effect of all included variables and their possible interactions, due to intrinsic computational restrictions of these models.

We validated the CI tree analysis findings by applying random forest modelling on the same data set. A benefit of applying random forest modelling compared to using conventional models with relative risks or odds ratios is that it ranks the predictors according to how important these are for explaining the outcome. However, the random forest analysis does not provide information on whether the predictors have a positive or negative relation to the outcome, nor the position of a predictor in a particular tree.

Our finding that education is important for development and welfare is perhaps a truism but recently covered in a chapter in a book on social progress for the twenty-first century by Spiel et al. [18] and with special emphasis on education’s role in low- and middle-income countries’ development by Abdi and Guo [19].

That the non-randomized interventions (water installation, microcredits, and participation in educational activities) positively influenced welfare found in the CI tree confirms our earlier results [8]. A recent publication tried to accomplish comparisons for the Millennium development villages evaluation [20], and the Randomized Controlled Trial (RTC) evaluation of multifaceted programs in six countries have comparison villages [21], both reports show positive results for complex interventions aiming for increased welfare in poor areas. The case study the Cuatro Santos experience constitute [7] has no comparison area so we cannot rule out that the general transformation of the Nicaraguan society is a reason for the improvements in welfare seen in the area. It is necessary to add that the Cuatro Santos case as a bottom-up, locally driven effort to increase welfare cannot offer the randomization of interventions that the scientific rigor for RCTs requires. One might even question if producing scientific proof of such quality is the best road to achieve positive change and development towards welfare. Perhaps social change is better achieved by examples from different context’s, considering the variation in different areas and let examples serve as an inspiration and not as recipes to follow. That the results are meaningful, comprehensible and showing a familiar truth about predictors of poverty in the area, was confirmed in a feedback and inference discussion held in the area with local community leaders and laypeople from different societal areas as health and security. The local community representatives confirmed the usefulness of this and similar further analyses for targeting interventions intending to reduce inequity. For such use, it is vital to bear in mind that detailed targeting could be stigmatizing.

## Conclusion

Predictors of poverty are useful for directing interventions; in the Cuatro Santos area, education seems most important to prioritize. The last decade’s general poverty reduction experienced in the study area does not cover the whole population, and further interventions increasing the educational level might yield further poverty reduction. However, the Nicaraguan social unrest during 2018 and its aftermath will most likely prevent further poverty reduction in general and specifically through developmental work as conducted in the Cuatro Santos case. This tragic development notwithstanding, hopefully, the lessons learned can continue to develop the Cuatro Santos communities as well as development in similar poor rural settings around the world.

## Supplementary information

**Additional file 1: Figure S1.** Conditional random forest plot ranking the relative importance (x-axis) of the 10 predictors with highest relative importance (y-axis) with regard to their ability to explain the presence of poverty in a household (2–4 unsatisfied basic needs) in Cuatro Santos, Nicaragua.

**Additional file 2: Figure S2.** Conditional random forest plot ranking the relative importance (x-axis) of the 10 predictors with highest relative importance (y-axis), when assets were removed as candidate predictors, with regard to their ability to explain the presence of poverty in a household (2–4 unsatisfied basic needs) in Cuatro Santos, Nicaragua.

## Data Availability

The datasets used and analyzed during the current study are available from the corresponding author on reasonable request.

## Abbreviations

CI: Conditional inference
CRF: Conditional random forest
HDSS: Health and demographic surveillance system
HFIAS: Household Food Insecurity Access Scale
MSE: Mean squared error
RTC: Randomized controlled trial
UBN: Unsatisfied basic need
