# Supplementary figures and images for: Predicting poverty. Data mining approaches to the health and demographic surveillance system in Cuatro Santos, Nicaragua
Source: Int J Equity Health. 2019 Oct 29;18:165. doi: 10.1186/s12939-019-1054-7 (PMC6819397; doi:10.1186/s12939-019-1054-7)

Additional figure 1


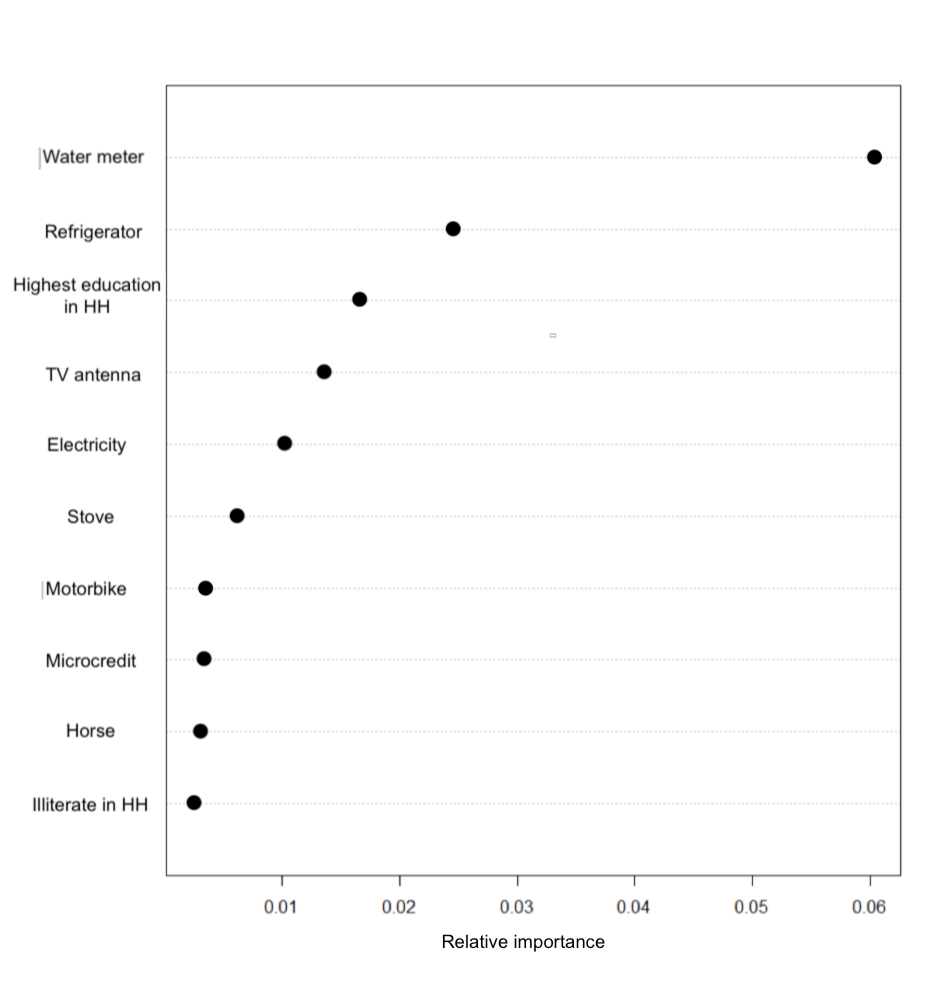

Supplement: Supplementary file 1 — Additional file 1: Figure S1. Conditional random forest plot ranking the relative importance (x-axis) of the 10 predictors with highest relative importance (y-axis) with regard to their ability to explain the presence of poverty in a household (2–4 unsatisfied basic needs) in Cuatro Santos, Nicaragua. [file 12939_2019_1054_MOESM1_ESM.docx]

Additional figure 2


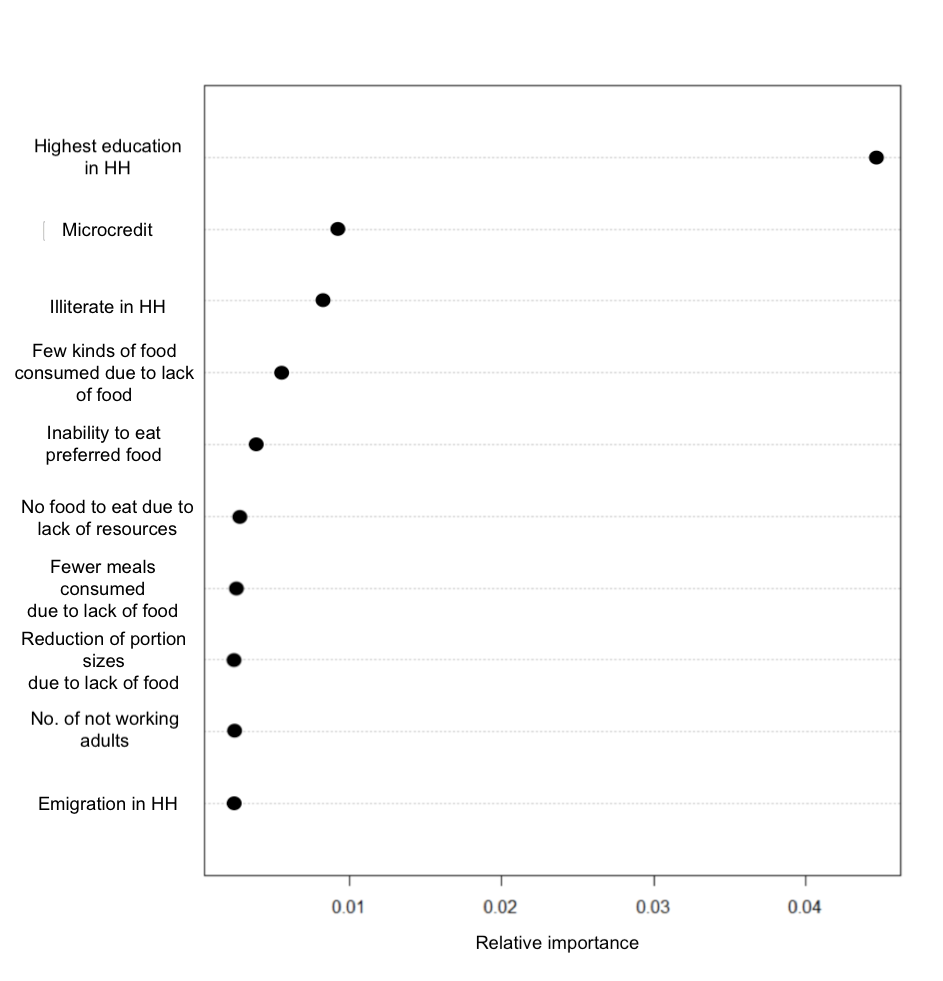

Supplement: Supplementary file 2 — Additional file 2: Figure S2. Conditional random forest plot ranking the relative importance (x-axis) of the 10 predictors with highest relative importance (y-axis), when assets were removed as candidate predictors, with regard to their ability to explain the presence of poverty in a household (2–4 unsatisfied basic needs) in Cuatro Santos, Nicaragua. [file 12939_2019_1054_MOESM2_ESM.docx]
